# 

**Published:** 1872-05

**Authors:** 


☞ The JOURNAL is published Monthly, at THREE DOLLARS PER ANNUM, in advance. Each Number contains Sixty-four Pages.

ATLANTA

### MEDICAL AND SURGICAL JOURNAL.

EDITED BY

JOSEPH P. LOGAN, M.D.,

AND

W. F. WESTMORELAND, M.D.,

*Professor of the Principles and Practice of Surgery in Atlanta Medical College.*

S.G.O.

VOL. X.—MAY, 1872.—NO. 2.

*PAX ET SCIENTIA, SED VERITAS SINE TIMORE.*

ATLANTA, GEORGIA:

PLANTATION PUBLISHING COMPANY'S PRESS.

1872.

